# Pneumococcal Serotypes Associated with Community-Acquired Pneumonia Hospitalizations in Adults in Spain, 2016–2020: The CAPA Study

**DOI:** 10.3390/microorganisms11112781

**Published:** 2023-11-16

**Authors:** Rosario Menéndez, Antoni Torres, Pedro Pablo España, Jose Alberto Fernández-Villar, José María Marimón, Raúl Méndez, Catia Cilloniz, Mikel Egurrola, Maribel Botana-Rial, María Ercibengoa, Cristina Méndez, Isabel Cifuentes, Bradford D. Gessner

**Affiliations:** 1 Hospital Universitario y Politécnico la Fe, 46026 Valencia, Spain; rosmenend@gmail.com (R.M.); rmendezalcoy@gmail.com (R.M.); 2 Biomedical Research Center Network for Respiratory Diseases (CIBERES), 28029 Madrid, Spain; 3 Hospital Clinic, 08036 Barcelona, Spain; catiacilloniz@yahoo.com; 4 Hospital Galdakao-Usansolo, 48960 Galdácano, Spain; pedropablo.espanayandiola@osakidetza.eus (P.P.E.); mikel.egurrolaizquierdo@osakidetza.eus (M.E.); 5 Hospital Alvaro Cunqueiro, Instituto de Investigación Biomédica Galicia Sur, 36312 Vigo, Spain; jose.alberto.fernandez.villar@sergas.es (J.A.F.-V.); maria.isabel.botana.rial@sergas.es (M.B.-R.); 6 Biodonostia, Hospital Universitario Donostia, 20014 San Sebastián, Spain; josemaria.marimonortizdezarate@osakidetza.eus (J.M.M.); maria.ercibengoaarana@biodonostia.org (M.E.); 7 Faculty of Health Sciences, Continental University, Huancayo 12001, Peru; 8 Pfizer S.L.U., 28108 Madrid, Spain; cristina.mendezdiez@pfizer.com (C.M.); isabel.cifuentesotero@pfizer.com (I.C.); 9 Pfizer Vaccines, Collegeville, PA 19426, USA; bradford.gessner@pfizer.com

**Keywords:** community-acquired pneumonia, pneumococcal conjugate vaccines, pneumococcal pneumonia, PCV20 serotypes

## Abstract

Newer higher valency pneumococcal conjugate vaccines (PCVs) have the potential to reduce the adult community-acquired pneumonia (CAP) burden. We describe the evolution and distribution of adult community-acquired pneumonia (CAP) serotypes in Spain, focusing on serotypes contained in the 20-valent PCV (PCV20). This was a prospective, observational study of chest X-ray (CXR)-confirmed CAP in immunocompetent adults hospitalized in one of four Spanish hospitals between November 2016 and November 2020. Pneumococci were isolated from cultures and detected in urine using BinaxNow^®^ and Pfizer serotype-specific urinary antigen tests UAD1 and UAD2. We included 1948 adults hospitalized with CXR-CAP. The median age was 69.0 years (IQR: 24 years). At least one comorbidity was present in 84.8% (*n* = 1653) of patients. At admission, 76.1% of patients had complicated pneumonia. Pneumococcus was identified in 34.9% (*n* = 680) of study participants. The PCV20 vaccine-type CAP occurred in 23.9% (*n* = 465) of all patients, 68.4% (*n* = 465) of patients with pneumococcal CAP, and 82.2% (83/101) of patients who had pneumococcus identified by culture. Serotypes 8 (*n* = 153; 7.9% of all CAP) and 3 (*n* = 152; 7.8% of all CAP) were the most frequently identified. Pneumococcus is a common cause of hospitalized CAP among Spanish adults and serotypes contained in PCV20 caused the majority of pneumococcal CAP.

## 1. Introduction

**Figure 1:**
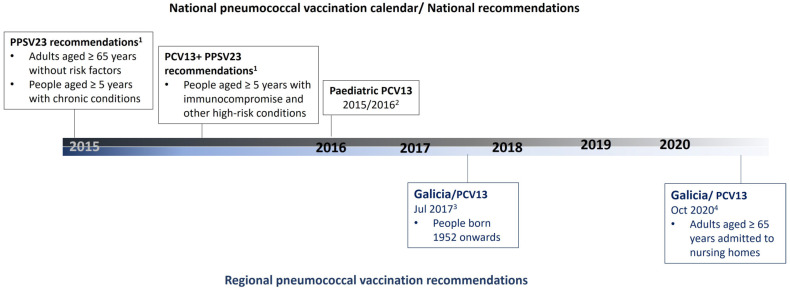
Summary of national and regional pneumococcal immunization recommendations during the study period in the areas included. 1. Ministerio de Sanidad, Servicios Sociales e Igualdad. Vacunación frente a neumococo en grupos de riesgo. Available at https://www.sanidad.gob.es/en/profesionales/saludPublica/prevPromocion/vacunaciones/programasDeVacunacion/docs/Neumococo_Gruposriesgo.pdf (accessed on 2 May 2015). 2. Ministerio de Sanidad Servicios Sociales e Igualdad. Revisión del calendario de vacunación. Ponencia de Programa y Registro de Vacunaciones. Available at https://www.sanidad.gob.es/profesionales/saludPublica/prevPromocion/vacunaciones/calendario-y-coberturas/docs/Revision_CalendarioVacunacion.pdf (accessed on 18 June 2022). 3. Consellería de Sanidade. Xunta de Galicia. Vacunación antipneumocócica en adultos 2017. Available at https://www.sergas.es/Saude-publica/Documents/4536/Nota_informativa_vacinacion_antipneumococica_2017.pdf (accessed on 21 May 2022). 4. Consellería de Sanidade. Xunta de Galicia. Nota informativa sobre a vacinación antipneumocócica conxugada en pacientes en residencias da terceira idade setembro 2020. Available at https://www.sergas.es/Saude-publica/Documents/6572/PNEUMO_CONXUGADA_RESIDENCIAS.pdf (accessed on 21 May 2015). PCV13: 13-valent pneumococcal conjugate vaccine; PPSV23: 23-valent pneumococcal polysaccharide vaccine.

The introduction of pneumococcal vaccines in childhood immunization programs has dramatically reduced pneumococcal disease due to direct and indirect effects [1,2,3,4]. In Spain, the 13-valent pneumococcal conjugate vaccine (PCV13) that includes serotypes 1, 3, 4, 5, 6A, 6B, 7F, 9V, 14, 18C, 19F, 19A, and 23F was introduced in children nationally by 2015–2016 [5]. For adults (Figure 1), national recommendations advise the use of the 23-valent pneumococcal polysaccharide (PPSV23) for immunocompetent adults aged ≥ 65 years and for people aged ≥ 2 years with comorbid conditions [5]. For high-risk adults aged ≥ 18 years, it is recommended to use PCV13 followed by PPSV23. However, during the last 6 years, several autonomous regions in Spain have also introduced PCV13 in specific groups of adults, e.g., Galicia in 2020 in adults aged ≥ 65 years admitted to nursing homes [6] and Valencia in 2021 in the same population [7]).

Pneumococcal vaccination policies for infants have substantially reduced PCV13 vaccine-type (PCV13-VT) pneumococcal disease in both children and adults [2,8]. Nevertheless, a certain burden of disease due to vaccine serotypes persists in adult populations, suggesting a plateau in the indirect protection provided by childhood vaccination [2,9]. Additionally, a large proportion of CAP in adults is attributed to serotypes not covered by PCV13 vaccine types (non-PCV13-VT) [2,9,10,11], serotypes 8, 22F, and 11A being the most frequently identified in Spain [2,10,11].

The 20-valent PCV (PCV20) is the highest valency PCV currently licensed by the European Medicines Agency (EMA) for the prevention of invasive disease and pneumonia caused by *Streptococcus pneumoniae* in individuals 18 years of age and older [12]. PCV20 includes PCV13 serotypes plus seven additional serotypes (8, 10A, 11A, 12F, 15B, 22F, 33F) [12] that have been commonly associated with invasive pneumococcal disease (IPD) [2,13] and linked to antibiotic resistance or greater disease severity [14,15,16,17].

A multicentre, observational, prospective, hospital-based study was conducted from 2011 to 2020 to determine the contribution of specific serotypes to CAP in immunocompetent adults. In this paper, we focus on the results of the last four years (2016–2020) and evaluate changes in serotype distribution, focusing primarily on PCV20-VTs and their comparison with other currently approved pneumococcal vaccines, and the association between PCV20-VTs and the presence of comorbidities and disease severity.

## 2. Materials and Methods

### 2.1. Participants

The study design and methodology have been previously described [10,18]. Immunocompetent adults aged 18 years or older with CAP admitted between November 2011 and November 2020 to any of the following Spanish tertiary hospitals were included in the study: Hospital Galdakao-Usansolo, Bizkaia (Basque country), Hospital Clinic I Provincial, Barcelona (Catalonia), Hospital La Fe, Valencia (Valencia), and Hospital Álvaro Cunqueiro, Pontevedra (Galicia). The study protocol was approved by the ethics committee of each of the hospitals, and centrally approved by the Ethical Committee of Hospital Clinic (Barcelona), with a registration number of 2011/6799. All the participants gave written informed consent. We report data from the period 2016–2020 (Supplementary Figure S1). Inclusion criteria, microbiological studies, and definitions of complicated CAP are detailed in Supplementary Material and previous publications [10,19,20,21].

### 2.2. Study Procedures

Demographic information, medical history, and clinical data of participants (including underlying conditions, tobacco cigarette smoking, alcohol consumption, history of previous pneumonia, history of hospitalization for at least 48 h within the 2 weeks before current admission, and pneumococcal and influenza vaccination history) were recorded as per standard clinical practice. Severity at the time of hospital admission was analysed by the CURB-65 score, Pneumonia Severity Index (PSI), and the Infectious Diseases Society of America/American Thoracic Society (IDSA/ATS) minor criteria. Patient evolution and outcome were also collected.

### 2.3. Microbiological Assessments

Microbiological tests were performed according to standard clinical practice [19]. High-quality sputum was defined as <10 epithelial cells and >25 leukocytes per field with 100× magnification. The sputum cultures with exclusive or abundant *S. pneumoniae* growth and consistent Gram staining were considered positive for pneumococcus. Urine samples were collected from all the participants (*n* = 1948). Urine specimens (*n* = 1782) were tested using the BinaxNow^®^ *S. pneumoniae* Urinary Antigen Test and BinaxNow^®^ *Legionella pneumophila* Urinary Antigen Test.

Serotype-specific urinary antigen detection (UAD) tests UAD1 and UAD2 were used in all the urine samples (*n* = 1948) to detect serotypes contained in the 20-valent PCV and serotypes 2, 9N, 17F, and 20 [22,23]. Serotyping was also performed on isolates from blood or pleural fluid cultures. The isolates and urine samples were processed as described in Torres et al. 2021 [10].

### 2.4. Definitions

Pneumococcal CAP was defined as chest X-ray (CXR)-confirmed CAP with a positive result from any microbiological test for *S. pneumoniae* (BinaxNow^®^ test, UAD1/UAD2 tests, blood culture, pleural fluid culture, and sputum culture). CXR confirmation was based on clinical judgement. Invasive pneumococcal CAP was defined as the isolation of *S. pneumoniae* from blood or pleural fluid culture. Non-invasive pneumococcal CAP was defined as confirmed pneumococcal CAP with positive UAD1/UAD2 results and negative blood and/or pleural fluid culture results. Pneumococcal serotypes were categorized as PCV13-VT: 1, 3, 4, 5, 6A, 7F, 6B, 9V, 14, 18C, 19A, 19F, and 23F; PCV15-VT: PCV13-VT plus 22F and 33F; PCV20-VT: PCV15-VT plus 8, 10A, 11A, 12F, and 15 B/C; PPSV23-VT: PCV20-VT plus 2, 9N, 17F, and 20, but minus 6A.

Complicated CAP was defined as the presence of one of the following on admission: bacteraemia, multilobar infiltrates, pleural effusion or empyema, respiratory failure, sepsis, and/or septic shock. Treatment failure was considered according to Spanish guidelines [19]. Clinical stability with modified Halm criteria was achieved when the patient’s vital signs were stable for a 24 h period, i.e., heart rate < 100 beats per minute; respiratory rate < 24 breaths per minute; axillary temperature < 37.2 °C; systolic blood pressure > 90 mmHg; oxygen saturation > 90% while breathing room air or at baseline for patients with chronic obstructive lung disease or those receiving oxygen therapy at home; good level of consciousness; tolerance to oral intake; adequate hydration and nutrition; and the absence of other active clinical or psychosocial problems requiring hospitalization [21].

### 2.5. Data Analyses

The baseline demographics and clinical data were summarized using the means and standard deviation, or the medians and interquartile range, as appropriate. The Pearson chi-squared test (or the exact Fisher test for 2 × 2 tables or likelihood ratio for MxN tables) was performed to compare proportions. For comparisons of independent groups, the Student’s *t*-test (or its non-parametric equivalent Mann–Whitney U test), and the single factor ANOVA and Kruskal–Wallis H test for continuous variables were used. Assumptions of normality and homoscedasticity of the variables were studied for the use of parametric tests. Furthermore, exploratory bivariate logistic regression models were used to explore how each identified serotype influences intensive care unit (ICU) admission, severity of disease or death. Severity/complications of pneumococcal pneumonia were used as the dependent variable and the identified serotype compared to no serotype was identified as the independent variable. The R software V 4.1.1, EPIDAT 3.0, and SPSS 19.0 software were used to analyse the data.

## 3. Results

### 3.1. Participants

During 2016–2020, 2164 participants were enrolled, including 1948 evaluable participants (Supplementary Figure S1). The median patient age was 69.0 years (Interquartile range [IQR]: 24 years), and 60.3% were men (Table 1).

### 3.2. Clinical Outcomes

At hospital admission, 76.1% of patients (*n* = 1482) had complicated pneumonia, most commonly acute respiratory failure (17.4%), sepsis (16.5%), and multilobar involvement (11.1%). During hospitalization, 700 patients (35.9%) had at least one complication, most frequently acute respiratory failure (46.4%), pleural effusion (28.1%), and acute renal failure (12.4%). The median length of hospital stay (LOS) was 7.0 days (IQR: 5.0 days) while the median length of ICU stay was 6 days (IQR: 6 days). In-hospital plus 30-day case fatality ratio (CFR) occurred in 2.2% (*n* = 42) of patients.

### 3.3. Serotype Distribution

The culture was performed in blood samples (*n* = 1298), respiratory samples (high-quality sputum *n* = 826), and pleural fluid samples (*n* = 150). *S. pneumoniae* was identified using diagnostic testing of any type in 34.9% (*n* = 680) of patients. Serotypes included in PCV20 were identified in 23.9% (*n* = 465) of all the CAP cases (Table 2). Serotype 8 was the most frequently identified serotype during the study period (7.9%; *n* = 153), followed by serotype 3 (7.8%; *n* = 152). Among patients with pneumococcal CAP, serotypes included in PCV20 accounted for 68.4% of cases (*n* = 465) over the study period.

Non-invasive/non-bacteraemic pneumococcal CAP was identified in 579 patients (29.7% of all CAP and 85.1% of pneumococcal CAP cases); the most common serotypes were 3 (23.8%; *n* = 138) and 8 (19.3%; *n* = 112). Invasive/bacteraemic pneumococcal CAP was identified in 101 patients (5.2% of all CAP and 14.9% of pneumococcal CAP); the most frequently identified serotypes were 8 (40.6%; *n* = 41) and 3 (13.9%; *n* = 14). Among invasive pneumococcal CAP cases, PCV20 serotypes tended to increase from 2016–2017 to 2019–2020 (*p* = 0.012) (see Table 2). Due to a lack of samples or lysis, four of the invasive pneumococcal pneumonia isolates could not be serotyped.

### 3.4. Patient Characteristics by Serotype

**Figure 2:**
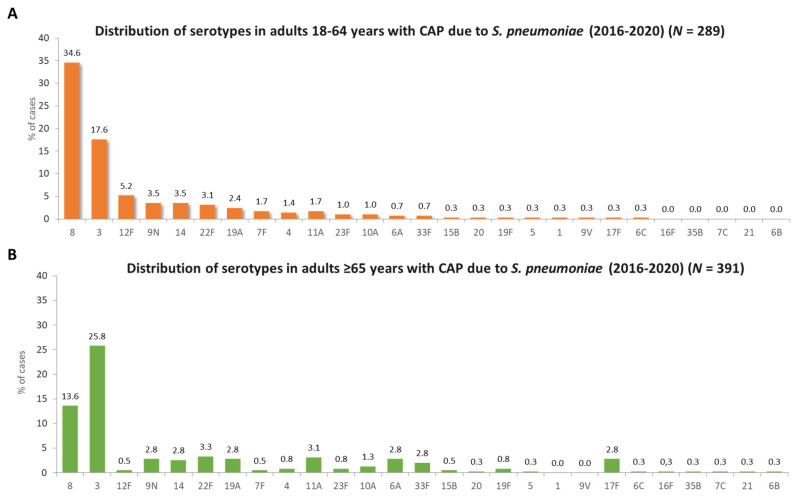
Distribution of serotypes in hospitalized adults with pneumococcal pneumonia in Spain between 2016–2020 aged 18–64 years (*N* = 289) (**A**) or ≥65 years (*N* = 391) (**B**).

The PCV20 serotypes accounted for a higher proportion of all CAP among patients aged 18–64 years than in patients aged ≥ 65 years (27.6% vs. 21.2%, *p* = 0.001) (Table 3). Among individual serotypes, serotypes 3 and 17F were more frequent in patients aged ≥ 65 years than in younger patients (*p* = 0.048 and *p* = 0.02, respectively). Serotypes 8 and 12F were more common among patients aged 18–64 years than in older patients (*p* < 0.001 for both serotypes). Figure 2 shows the distribution of specific serotypes in patients with pneumococcal CAP by age group. In both age groups, serotypes 8 and 3 were the most frequent, although eight was more common in patients aged < 65 years compared to those 65 years or older (34.6% vs. 13.5%, *p* < 0.001).

Serotype distribution by severity on admission is shown in Supplementary Table S1. Patients with complicated pneumonia had a higher percentage of serotypes included in PCV20 than those with non-complicated pneumonia (25.1% vs. 20.1%, *p* = 0.027). Serotype 3 was more frequently identified in participants with complicated pneumonia compared to those with non-complicated pneumonia on hospital admission (8.7% vs. 5.0%, *p* = 0.009).

Similar rates of CAP caused by vaccine-type (VT) serotypes were observed in adults with no comorbidities and with at least one comorbidity. Nevertheless, for adults with comorbidities, the percentage of VT cases differed by number and type of underlying diseases (Table 4 and Supplementary Table S2).

When VT serotypes were analysed by the presence of underlying conditions and age group (Supplementary Table S3), we found that among patients with chronic obstructive pulmonary disease (COPD), PCV20-VT serotypes were significantly more frequent in younger patients (aged 18–64 years) than in those aged 65 years or older (36.4% vs. 20.4%, *p* = 0.009). Similar findings were observed in patients with diabetes and chronic renal failure. These differences were due mainly to the higher percentage of cases caused by serotypes 3 and 8 in younger patients with comorbidities.

A comparison of the evolution of pneumococcal serotypes in two locations with different pneumococcal vaccination policies (Catalonia and Galicia) is shown in Table 5 [6,24,25]. Hospital Clinic is located in Catalonia, a region that follows national recommendations and advice for the use of the PPV23 vaccine in adults aged at least 65 years old or in adults with underlying conditions and where PCV13 was not introduced in the immunization program for children until 2016. Hospital Álvaro Cunqueiro is located in Galicia, a region that introduced vaccination with the PCV13 vaccine in adults aged 65 years in July 2017 and where PCV13 was included in the childhood immunization program in 2011 [6,24,25]. The proportion of cases due to PCV20-VT was similar in both regions (24.5% in Catalonia vs. 24.9% in Galicia), although there were significant differences in the total number of all CAPs in which serotypes 14 and 8 were identified (2.5% vs. 0.2%, respectively, *p* = 0.002; and 4.3% and 10.8%, respectively, *p* = 0.001).

### 3.5. Clinical Outcomes by Serotype

Supplementary Tables S4 and S5 show serotype distribution by complications and ICU admission during hospitalization, respectively. PCV20-VTs were slightly more common in CAP patients developing complications during hospitalization (27.7% vs. 21.7%, *p* = 0.003) and substantially more common among patients admitted to ICU (34.4% vs. 22.6%, *p* < 0.001, respectively). Serotype 3 was the most common individual serotype identified in patients with complications (10.0%) or admitted to ICU (16.5%).

The in-hospital plus 30-day CFR was 2.2% for all the CAP cases. Of 42 fatal cases, the most common serotypes were 3 (9.5%; *n* = 4), 8, 14, 19A, 17F, and 22F (2.4% each; *n* = 1). We did not calculate serotype-specific CFR due to the low numbers of deaths within individual serotypes.

**Figure 3:**
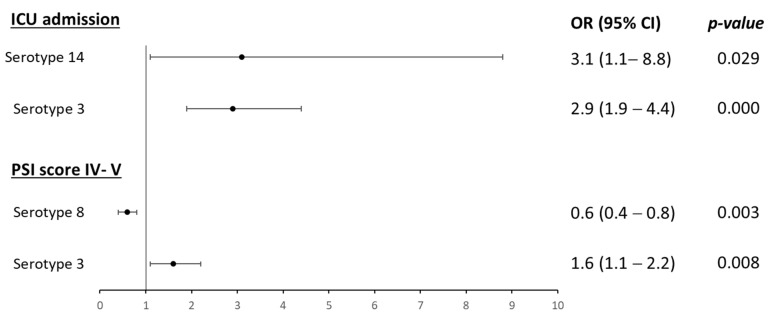
Serotypes associated with severity on admission or during hospitalization in bivariate analysis Only serotypes with significant values are shown in the figure. For comparisons, no serotype identified is the reference parameter. ICU: intensive care unit; OR: odds ratio; PSI: pneumonia severity index.

In the bivariate logistic regression models, both serotypes 3 and 14 were associated with higher odds of ICU admission (OR 2.9, 95% CI 1.9–4.4, and OR 3.1, 95% CI 1.1–8.8, respectively) (Figure 3). Serotype 8 was associated with a lower pneumonia severity index (PSI) on admission (OR 0.6, 95% CI 0.4–0.8), while serotype 3 was associated with a higher PSI (OR 1.6, 95% CI 1.1–2.2)**.**

## 4. Discussion

We identified *S. pneumoniae* in 35% of immunocompetent hospitalized patients with CAP in Spain between 2016 and 2020, while 24% of all the CAP cases were due to pneumococcal serotypes included in PCV20, 12% to serotypes included in PCV13, 14% to serotypes included in PCV15, and 25% to PPSV23 serotypes. The PCV20 serotypes were more common than other serotypes among individuals younger than 65 years old and lead to invasive disease, complications at hospital admission, and ICU admission. The most frequent serotypes found in our study were 3 and 8, followed by 22F.

In Spain, the PCV13 introduction in the childhood National Immunization Program has been linked to quick and sustained decreases in total and VT IPDs in young children and older unvaccinated cohorts [2]. Recent data, however, indicate that the indirect effects have plateaued in many regions, including those with great coverage, specifically for serotypes 3, 7F, 19A, and 19F [26]. Thus, the prevalence of PCV13 serotypes in Spanish adults showed that childhood vaccination provided insufficient indirect protection [10]. Moreover, non-PCV13 serotypes, especially serotype 8, have a rising trend in the country [2]. Our study describes the evolution and distribution of pneumococcal serotypes causing CAP in adults, focusing on PCV20, the highest valency PCV currently licensed, which includes serotype 8 and could help reduce the CAP burden in adult populations.

We observed a rise in the number of CAP cases detected due to *S. pneumoniae* in Spain during the last four years, compared to our previously published data (35% vs. 29%) [10]. The introduction of the UAD-2 diagnostic test, which detects serotypes 2, 8, 9N, 10A, 11A, 12F, 15B, 17F, 20, 22F, and 33F, might be the main reason for this apparent increase since it is more sensitive for identifying pneumococcal serotypes than either blood culture or BinaxNow^®^ while maintaining specificity [23]. The improved diagnostic sensitivity for the non-bacteraemic CAP suggests that our results more accurately reflect the burden of pneumococcal CAP. The inclusion of additional serotypes in future iterations of the UAD should further improve the pneumococcal CAP burden estimates.

Several studies from Spain and other European countries, such as the United Kingdom, reported similar proportions of CAP due to *S. pneumoniae* [11,27]. In contrast, a lower proportion of the CAP cases associated with *S. pneumoniae* was reported from two studies in the USA (9.9% and 12.3%), and Greece (13.5%), even though they also used UAD1 and UAD2 [9,28,29]. These differences could be partially explained by the pneumococcal vaccination policy for children and adults. As in most other European countries, Spain recommends a 2 + 1 PCV13 schedule for children. During the study period, PPSV23 was preferentially recommended in Spain for adult immunization. In Greece, the paediatric immunization program included PCV13 in a 3 + 1 schedule until June 2019 and 2 + 1 thereafter, and uses both PCV13 and PPSV23 for adult immunization, while the USA has consistently recommended a 3 + 1 schedule for children and, since 2014, PCV13 for adults aged 65 years and older [30]. Hence, it is possible that patients in Greece and the USA had improved herd immunity as a result of the 3 + 1 infant schedule or better direct protection because of the PCV13 vaccination of older adults.

Serotype 3 remains one of the most common serotypes in Spain. Numerous studies have found that PCV13 provides protection against IPD and CAP among directly vaccinated persons [31]. However, its ability to reduce carriage may be lower than for other vaccine serotypes, thus offering less or no indirect protection from childhood immunization [32]. Moreover, a new serotype 3 clade with high levels of antibiotic resistance and altered subcapsular proteins may increase community transmission and raise the risk of disease, particularly among unvaccinated individuals [33,34]. In Spain, serotype 3 disease risk among older adults was probably compounded by low pneumococcal vaccination coverage (including with PCV13) for this target group during the study period [2].

As in other European sites [35], the rising trend previously observed in Spain for serotype 8 was maintained [10]. In Spain, serotype 8 is mostly associated with two main clonal complexes (CC53 and CC404), both related to penicillin-susceptible isolates [36]. The fact that this serotype was less frequent among older adults may be associated with this antimicrobial susceptibility pattern, as antimicrobial-resistant pneumococci appear more frequently in older patients with comorbidities [11]. Previous studies have linked the presence of serotype 8 to highly invasive disease and moderate CFR [15]. If Spain implements PCV20 among older adults and young children, the degree to which serotype 8 is reduced will depend on serotype-specific efficacy against disease and carriage, as well as vaccine uptake, and will require ongoing monitoring of IPD and CAP, including non-bacteraemic CAP.

In our study, we identified similar numbers of CAP cases among persons aged 18–64 years with comorbidities as in adults aged 65 years and older, and PCV20-VTs were more common in the former. This raises the possibility that new, higher valency PCVs will provide a similar public health benefit in both populations, a conclusion reached by the United States Advisory Committee on Immunization Practices (ACIP) when they recommended routine PCV immunization of both groups [37]

In our study, PCV20-VT serotypes were associated with more severe clinical manifestations, including invasive disease, complications during hospitalization, and ICU admission. Previous reports have also shown a correlation between serotypes included in PCV20 and increased disease severity [13,14,38,39]. This is to be expected, as the serotypes in PCV13 and subsequently, PCV20 were chosen in part for their association with IPD and severe disease [13,38,39,40].

This investigation has some limitations, as described in the previously published results of the CAPA study [10]. Firstly, the proportion of the CAP cases due to vaccine serotypes may have been overestimated, because no serotype-specific antigen exists for most serotypes not contained in PCV20. However, we found that the percentage of patients with pneumococcus infection identified by culture who had a PCV20-VT was 82.2% (83/101), indicating that the overestimation was not substantial. Moreover, the sensitivity of the UAD assays for non-bacteraemic pneumonia remains undefined due to the lack of a gold standard, and this value may be lower than that reported for bacteraemia pneumonia [41]. Furthermore, we did not calculate CAP incidence, and we could not identify the microorganism responsible for CAP in 49.3% of the cases. We only investigated the burden of CAP in hospitalized, immunocompetent patients, and the results may not be representative of the outpatient setting or immunocompromised patients. The characteristics of our patient population also may explain the relatively low mortality rate found in the study. Lastly, our study included data collected during the peak of the COVID-19 pandemic, during which non-pharmaceutical interventions, such as mask-wearing and restrictions on social mingling, may have altered serotype epidemiology in a way that is not reflective of non-COVID-19 periods; this possibility will need to be assessed with ongoing surveillance.

## 5. Conclusions

Pneumococcus is a common cause of CAP in older adults in Spain and PCV20-VTs are a common cause of pneumococcal CAP. Newer, expanded valency PCVs, particularly those that contain the highly prevalent serotype 8, have the potential to substantially reduce disease burden. The specific target groups for adult vaccination could be defined by formal cost-effectiveness analyses.

## Figures and Tables

**Table 1:** Characteristics of the study population in all community-acquired pneumonia cases, 2016–2020.

	All CAP (*n* = 1948)	CAP Due to *S. pneumoniae* (*n* = 680)
	*n* (%)	*n* (%)
**Age**		
18–64 years	801 (41.1)	289 (42.5)
≥65 years	1147 (58.9)	391 (57.5)
Median (IQR)	69.0 (24.0)	68.0 (23.0)
Male gender	1175 (60.3)	404 (59.4)
**≥** **1 underlying condition**	1652 (84.8)	582 (85.6)
**≥** **2 underlying conditions**	1107 (67.0)	371 (54.6)
COPD	351 (18.0)	119 (17.5)
Chronic heart failure	183 (9.4)	50 (7.4)
Diabetes mellitus	423 (21.7)	138 (20.3)
Chronic liver disease	59 (3.0)	29 (4.3)
Chronic renal failure	168 (8.6)	43 (6.3)
Stroke	135 (6.9)	45 (6.6)
Asthma	204 (10.5)	79 (11.6)
Cured cancers	207 (10.6)	73 (10.7)
Previous hospitalization	60 (3.1)	21 (3.1)
Previous pneumonia	360 (18.5)	107 (15.7)
Tobacco cigarette smoking ^a^	405 (20.8)	159 (23.4)
Alcoholism ^b^	68 (3.5)	29 (4.3)
**Vaccination history**		
Influenza vaccine	1022 (52.5)	335 (49.3)
Pneumococcal vaccine	424 (21.8)	134 (19.7)
PPSV23 ^c^	347 (81.6)	111 (82.2)
18–64 years	37 (10.7)	9 (8.1)
≥65 years	310 (89.3)	102 (91.9)
PCV13 ^d^	94 (22.2)	38 (28.4)
18–64 years	15 (16.0)	6 (15.8)
≥65 years	79 (84.0)	32 (84.2)
Both	46 (10.8)	20 (14.8)
18–64 years	4 (8.7)	1 (5.0)
≥65 years	42 (91.3)	19 (95.0)
**Severity on admission**		
PSI score IV–V	704 (36.1)	267 (39.3)
CURB-65 risk score 3–5	277 (14.2)	119 (17.5)
≥3 IDSA/ATS minor criteria for severe CAP	208 (10.7)	83 (12.2)
**Outcomes**		
ICU admission	218 (11.2)	92 (13.5)
In-hospital case fatality ratio	32 (1.6)	11 (1.6)
In-hospital plus 30-day case fatality ratio	42 (2.2)	12 (1.8)

^a^ Tobacco cigarette smoking: smokers of ≥10 cigarettes per day within the previous year or who quit smoking less than 6 months previously. ^b^ Alcoholism: intake ≥ 80 gr per day during at least the previous year. ^c^ 23-valent pneumococcal polysaccharide vaccine (1, 2, 3, 4, 5, 6B, 7F, 8, 9N, 9V, 10A, 11A, 12F, 14, 15B, 17F, 18C, 19A, 20, 22F, 23F, 33F serotypes). ^d^ 13-valent pneumococcal conjugate vaccine (1, 3, 4, 5, 6A, 6B, 7F, 9V, 14, 18C, 19A, 19F, 23F serotypes). COPD: chronic obstructive pulmonary disease; CURB-65: confusion, uremia, respiratory rate, BP, age ≥ 65 years; ICU: intensive care unit; IDSA/ATS: Infectious Diseases Society of America/American Thoracic Society; IQR: interquartile range; PCV13: 13-valent pneumococcal conjugate vaccine; PPSV23: 23-valent pneumococcal polysaccharide; PSI: pneumonia severity index; SD: standard deviation.

**Table 2:** Distribution of vaccine serotypes in all community-acquired pneumonia and pneumococcal community-acquired pneumonia by study period, 2016–2020.

	2016–2017		2017–2018		2018–2019		2019–2020		Total	
									(2016–2020)	
	*N*	%	*N*	%	*N*	%	*N*	%	*N*	%
**All CAP**	507		514		545		382		1948	
PCV13 serotypes	64	12.6	68	13.2	65	11.9	38	9.9	235	12.1
PCV15 serotypes	73	14.4	75	14.6	77	14.1	42	11.0	267	13.7
PCV20 serotypes	113	22.3	130	25.3	145	26.6	77	20.2	465	23.9
PPSV23 serotypes	119	23.5	144	28.0	142	26.1	82	21.5	487	25.0
**Most prevalent serotypes (≥1% in total cases)**										
8	32	6.3	39	7.6	50	9.2	32	8.4	153	7.9
3	40	7.9	45	8.8	42	7.7	25	6.5	152	7.8
22F	8	1.6	4	0.8	6	1.1	4	1.0	22	1.1
9N	5	1.0	10	1.9	2	0.4	4	1.0	21	1.1
14	6	1.2	5	1.0	6	1.1	3	0.8	20	1.0
**CAP due to *S. pneumoniae***	167	32.9	180	35.0	203	37.2	130	34.0	680	34.9
PCV13 serotypes	64	38.3	68	37.8	65	32.0	38	29.2	235	34.6
PCV15 serotypes	73	43.7	75	41.7	77	37.9	42	32.3	267	39.3
PCV20 serotypes	113	67.7	130	72.2	145	71.4	77	59.2	465	68.4
PPSV23 serotypes	119	71.3	144	80.0	142	70.0	82	63.1	487	71.6
**Non-invasive CAP ^1^**	136	81.4	155	86.1	175	86.2	113	86.9	579	85.1
PCV13 serotypes	54	39.7	64	41.3	60	64.3	32	28.3	210	36.3
PCV15 serotypes	61	44.9	70	45.2	71	40.6	35	31.0	237	40.9
PCV20 serotypes	90	66.2	112	72.3	120	68.6	60	53.1	382	66.0
PPSV23 serotypes	95	69.9	121	78.1	116	66.3	65	57.5	397	68.6
**Invasive CAP ^2^**	31	18.6	25	13.9	28	13.8	17	13.1	101	14.9
PCV13 serotypes	10	32.3	4	16	5	17.9	6	35.3	25	24.8
PCV15 serotypes	12	38.7	5	20.0	6	21.4	7	41.2	30	29.7
PCV20 serotypes	23	74.2	18	72	25	89.3	17	100	83	82.2
PPSV23 serotypes	24	77.4	23	92.0	26	92.9	17	100	90	89.1

^1^ Confirmed pneumococcal CAP (according to UAD1/UAD2 or BinaxNow^®^tests) for which blood and/or pleural fluid culture results were negative; ^2^ *S. pneumoniae* isolated in blood and/or pleural fluid culture. Among 101 cases identified, 4 isolates were not serotyped. CAP: community-acquired pneumonia; PCV13: 13-valent pneumococcal conjugate vaccine; PCV15: 15-valent pneumococcal conjugate vaccine; PCV20: 20-valent pneumococcal conjugate vaccine; PPSV23: 23-valent pneumococcal polysaccharide vaccine.

**Table 3:** Vaccine serotype distribution in all community-acquired pneumonia and pneumococcal community-acquired pneumonia by age group and study period, 2016–2020.

	18–64 Years					≥65 Years					*p*-Value
	2016–2017	2017–2018	2018–2019	2019–2020	Total	2016–2017	2017–2018	2018–2019	2019–2020	Total	
	*N* (%)	*N* (%)	*N* (%)	*N* (%)	*N* (%)	*N* (%)	*N* (%)	*N* (%)	*N* (%)	*N* (%)	
**All CAP**	**174**	**227**	**238**	**162**	**801**	**333**	**287**	**307**	**220**	**1147**	
PCV13 serotypes	21 (12.1)	29 (12.8)	20 (8.4)	16 (9.9)	86 (10.7)	43 (12.9)	39 (13.6)	45 (14.7)	22 (10.0)	149 (12.9)	0.133
PCV15 serotypes	23 (13.2)	30 (13.2)	26 (10.9)	18 (11.1)	97 (12.1)	50 (15.0)	45 (15.7)	51 (16.6)	24 (10.9)	170 (14.8)	0.087
PCV20 serotypes	45 (25.9)	66 (29.1)	70 (29.4)	40 (24.7)	221 (27.6)	68 (20.4)	64 (22.3)	75 (24.4)	37 (16.8)	244 (21.2)	0.001
PPSV23 serotypes	47 (27.0)	72 (31.7)	71 (29.8)	41 (25.3)	231 (28.8)	72 (21.6)	72 (25.1)	71 (23.1)	41 (18.6)	256 (22.3)	0.001
**Most prevalent serotypes** (≥1% of total cases) *											
8	19 (10.9)	27 (11.9)	34 (14.3)	20 (12.3)	100 (12.5)	13 (3.9)	12 (4.2)	16 (5.2)	12 (5.5)	53 (4.6)	<0.001
3	15 (8.6)	14 (6.2)	13 (5.5)	9 (5.6)	51 (6.4)	25 (7.5)	31 (10.8)	29 (9.4)	16 (7.3)	101 (8.8)	0.048
22F	2 (1.1)	1 (0.4)	4 (1.7)	2 (1.2)	9 (1.1)	6 (1.8)	3 (1.0)	2 (0.7)	2 (0.9)	13 (1.1)	1.000
14	2 (1.1)	4 (1.8)	3 (1.3)	1 (0.6)	10 (1.2)	4 (1.2)	1 (0.3)	3 (1.0)	2 (0.9)	10 (0.9)	0.495
9N	2 (1.1)	5 (2.2)	2 (0.8)	1 (0.6)	10 (1.2)	3 (0.9)	5 (1.7)	0 (0.0)	3 (1.4)	11 (1.0)	0.657
19A	1 (0.6)	4 (1.8)	2 (0.8)	0 (0.0)	7 (0.9)	5 (1.5)	1 (0.3)	4 (1.3)	1 (0.5)	11 (1.0)	1.000
12F	2 (1.1)	6 (2.6)	6 (2.5)	1 (0.6)	15 (1.9)	0 (0.0)	2 (0.7)	0 (0.0)	0 (0.0)	2 (0.2)	<0.001
17F	0 (0.0)	1 (0.4)	0 (0.0)	0 (0.0)	1 (0.1)	3 (0.9)	3 (1.0)	3 (1.0)	2 (0.9)	11 (1.0)	0.020
**CAP due to *S. pneumoniae***	58 (33.3)	83 (36.6)	92 (38.7)	56 (34.6)	289 (36.1)	109 (32.7)	97 (33.8)	111 (36.2)	74 (33.6)	391 (34.1)	
PCV13 serotypes	21 (36.2)	29 (34.9)	20 (21.7)	16 (28.6)	86 (29.8)	43 (39.4)	39 (40.2)	45 (40.5)	22 (29.7)	149 (38.1)	0.024
PCV15 serotypes	23 (39.7)	30 (36.1)	26 (28.3)	18 (32.1)	97 (33.6)	50 (45.9)	45 (46.4)	51 (45.9)	24 (32.4)	170 (43.5)	0.009
PCV20 serotypes	45 (77.6)	66 (79.5)	70 (76.1)	40 (71.4)	221 (76.5)	68 (62.4)	64 (66.0)	75 (67.6)	37 (50.0)	244 (62.4)	<0.001
PPSV23 serotypes	47 (81.0)	72 (86.7)	71 (77.2)	41 (73.2)	231 (79.9)	72 (66.1)	72 (74.2)	71 (64.0)	41 (55.4)	256 (65.5)	<0.001
**Non-invasive CAP ^1^**	47 (81.0)	67 (80.7)	71 (77.2)	46 (82.2)	231 (79.9)	89 (81.7)	88 (90.7)	104 (93.7)	67 (90.5)	348 (89.0)	
PCV13 serotypes	19 (40.4)	27 (40.3)	18 (25.4)	13 (28.3)	77 (33.3)	35 (39.3)	37 (42.0)	42 (40.4)	19 (28.4)	133 (38.2)	0.231
PCV15 serotypes	20 (42.6)	28 (41.8)	23 (32.4)	15 (32.6)	86 (37.2)	41 (46.1)	42 (47.7)	48 (46.2)	20 (29.9)	151 (44.4)	0.140
PCV20 serotypes	35 (74.5)	55 (82.1)	49 (69.0)	30 (65.2)	169 (73.9)	55 (61.8)	57 (64.8)	71 (68.3)	30 (44.8)	213 (61.2)	0.003
PPSV23 serotypes	36 (76.6)	57 (85.1)	50 (70.4)	31 (67.4)	174 (75.3)	59 (66.3)	64 (72.7)	66 (63.5)	34 (50.7)	223 (64.1)	0.004
**Invasive CAP ^2^**	11 (19.0)	16 (19.3)	21 (22.8)	10 (17.9)	58 (20.1)	20 (18.3)	9 (9.3)	7 (6.4)	7 (9.5)	43 (11.0)	
PCV13 serotypes	2 (18.2)	2 (12.5)	2 (9.5)	3 (30.0)	9 (15.5)	8 (40.0)	2 (22.2)	3 (42.9)	3 (42.9)	16 (37.2)	0.013
PCV15 serotypes	3 (27.3)	2 (12.5)	3 (14.3)	3 (30.0)	11 (19.0)	9 (45.0)	3 (33.3)	3 (42.9)	4 (57.1)	19 (44.2)	0.006
PCV20 serotypes	10 (90.9)	11 (68.8)	21 (100.0)	10 (100.0)	52 (89.7)	13 (65.0)	7 (77.8)	4 (57.1)	7 (100.0)	31 (72.1)	0.023
PPSV23 serotypes	11 (100.0)	15 (93.8)	21 (100.0)	10 (100.0)	57 (98.3)	13 (65.0)	8 (88.9)	5 (71.4)	7 (100.0)	33 (76.7)	0.001

^1^ Confirmed pneumococcal CAP (by UAD1/UAD2 or BinaxNow^®^ tests) for which blood and/or pleural fluid culture results were negative; ^2^ *S. pneumoniae* isolated in blood and/or pleural fluid culture. Among 101 cases identified, 4 isolates were not serotyped. * Calculated from the total number of cases by age group. CAP: community-acquired pneumonia; PCV13: 13-valent pneumococcal conjugate vaccine; PCV15: 15-valent pneumococcal conjugate vaccine; PCV20: 20-valent pneumococcal conjugate vaccine; PPSV23: 23-valent pneumococcal polysaccharide vaccine.

**Table 4:** Vaccine serotype distribution in all community-acquired pneumonia and pneumococcal community-acquired pneumonia according to the presence of at least one underlying condition, 2016–2020 ^a^.

		Diabetes Mellitus		COPD		Previous Pneumonia		Tobacco Cigarette Smoking		Chronic Heart Failure		Chronic Renal Failure		Asthma		No Underlying Diseases	
		*N*	%	*N*	%	*N*	%	*N*	%	*N*	%	*N*	%	*N*	%	*N*	%
**All CAP (*n* = 1948)**		**423**		**351**		**360**		**405**		**183**		**168**		**204**		**295**	
PCV13 serotypes		44	10.4	43	12.3	33	9.2	52	12.8	17	9.3	10	6.0	27	13.2	29	9.8
PCV15 serotypes		49	11.6	50	14.2	39	10.8	60	14.8	19	10.4	10	6.0	30	14.7	33	11.2
PCV20 serotypes		82	19.4	82	23.4	64	17.8	124	30.6	24	13.1	21	12.5	55	27.0	71	24.1
PPSV23 serotypes		95	22.5	90	25.6	69	19.2	130	32.1	26	14.2	20	11.9	54	26.5	74	25.1
Most prevalent serotypes (≥1% of total cases)	3	32	7.6	31	8.8	28	7.8	31	7.7	10	5.5	5	3.0	17	8.3	18	6.1
	8	426	6.1	19	5.4	21	5.8	50	12.3	4	2.2	8	4.8	23	11.3	30	10.2
	9N	9	2.1	6	1.7	4	1.1	7	1.7	1	0.5	1	0.6	2	1.0	2	0.7
	12F	1	0.2	3	0.9	0	0.0	5	1.2	0	0.0	2	1.2	1	0.5	4	1.4
	14	5	1.2	2	0.6	0	0.0	4	1.0	0	0.0	2	1.2	3	1.5	2	0.7
	17F	5	1.2	4	1.1	3	0.8	2	0.5	2	1.1	0	0.0	0	0.0	2	0.7
	19A	2	0.5	2	0.6	1	0.3	4	1.0	1	0.5	0	0.0	0	0.0	3	1.0
	22F	4	0.9	5	1.4	4	1.1	6	1.5	1	0.5	0	0.0	1	0.5	3	1.0
**CAP due to *S. pneumoniae*** **(*N* = 680; 34.9%)**		**138**		**119**		**107**		**159**		**50**		**43**		**79**		**98**	
PCV13 serotypes		44	31.9	43	36.1	33	30.8	52	32.7	17	34.0	10	23.3	27	34.2	29	29.6
PCV15 serotypes		49	35.5	50	42.0	39	36.4	60	37.7	19	38.0	10	23.3	30	38.0	33	33.7
PCV20 serotypes		82	59.4	82	68.9	64	59.8	124	78.0	24	48.0	21	48.8	55	69.6	71	72.4
PPSV23 serotypes		95	68.8	90	75.6	69	64.5	130	81.8	26	52.0	20	46.5	54	68.4	74	75.5
**Non-invasive CAP ^1^**		**122**		**99**		**91**		**127**		**40**		**37**		**66**		**82**	
PCV13 serotypes		41	33.6	36	36.4	31	34.1	46	36.2	11	27.5	9	24.3	22	33.3	26	31.7
PCV15 serotypes		44	36.1	40	40.4	35	38.5	52	40.9	13	32.5	9	24.3	25	37.9	30	36.6
PCV20 serotypes		70	57.4	64	64.6	55	60.4	94	74.0	18	45.0	18	48.6	42	63.6	57	69.5
PPSV23 serotypes		81	66.4	72	72.7	58	63.7	98	77.2	20	50.0	17	45.9	41	62.1	58	70.7
**Invasive CAP ^2^**		**16**		**20**		**16**		**32**		**10**		**6**		**13**		**16**	
PCV13 serotypes		3	18.8	7	35.0	2	12.5	6	18.8	6	60.0	1	16.7	5	38.5	3	18.8
PCV15 serotypes		5	31.3	10	50.0	4	25.0	8	25.0	6	60.0	1	16.7	5	38.5	3	18.8
PCV20 serotypes		12	75.0	18	90.0	9	56.3	30	93.8	6	60.0	3	50.0	13	100.0	14	87.5
PPSV23 serotypes		14	87.5	18	90.0	11	68.8	32	100.0	6	60.0	3	50.0	13	100.0	16	100.0

^a^ Patients might have more than one underlying condition. ^1^ Confirmed pneumococcal CAP (according to UAD1/UAD2 or BinaxNow^®^ tests) for which blood and/or pleural fluid culture results were negative; ^2^ *S. pneumoniae* isolated in blood and/or pleural fluid culture. Four isolates of invasive pneumococcal pneumonias were not serotyped due to lack of sample or lysis. CAP: community-acquired pneumonia; COPD: chronic obstructive pulmonary disease; PCV13: 13-valent pneumococcal conjugate vaccine; PCV15: 15-valent pneumococcal conjugate vaccine; PCV20: 20-valent pneumococcal conjugate vaccine; PPSV23: 23-valent pneumococcal polysaccharide vaccine.

**Table 5:** Distribution of vaccine serotypes in two participating centres, 2016–2020.

	Hospital Clinic, Barcelona (Catalonia)					Hospital Alvaro Cunqueiro (Galicia)					*p*-Value
	2016–2017	2017–2018	2018–2019	2019–2020	Total	2016–2017	2017–2018	2018–2019	2019–2020	Total	
	*N* (%)	*N* (%)	*N* (%)	*N* (%)	*N* (%)	*N* (%)	*N* (%)	*N* (%)	*N* (%)	*N* (%)	
**All CAP**	**66**	**70**	**104**	**37**	**277**	**151**	**157**	**148**	**138**	**594**	
PCV13 serotypes	11 (16.7)	9 (12.9)	20 (19.2)	7 (18.9)	47 (17.0)	19 (12.6)	22 (14.0)	10 (6.8)	15 (10.9)	66 (11.1)	0.017
PCV15 serotypes	11 (16.7)	10 (14.3)	23 (22.1)	7 (18.9)	51 (18.4)	20 (13.2)	22 (14.0)	11 (7.4)	15 (10.9)	68 (11.4)	0.005
PCV20 serotypes	17 (25.8)	13 (18.6)	29 (27.9)	9 (24.3)	68 (24.5)	31 (20.5)	40 (25.5)	44 (29.7)	33 (23.9)	148 (24.9)	0.907
PPSV23 serotypes	17 (25.8)	14 (20.0)	30 (28.8)	10 (27.0)	71 (25.6)	33 (21.9)	44 (28.0)	44 (29.7)	35 (25.4)	156 (26.3)	0.843
Most prevalent serotypes (≥1% in total cases) *											
14	2 (3.0)	3 (4.3)	2 (1.9)	0 (0.0)	7 (2.5)	1 (0.7)	0 (0.0)	0 (0.0)	0 (0.0)	1 (0.2)	0.002
19A	1 (1.5)	1 (1.4)	2 (1.9)	0 (0.0)	4 (1.4)	1 (0.7)	3 (1.9)	1 (0.7)	1 (0.7)	6 (1.0)	0.734
3	7 (10.6)	3 (4.3)	13 (12.5)	5 (13.5)	28 (10.1)	16 (10.6)	13 (8.3)	8 (5.4)	11 (8.0)	48 (8.1)	0.323
8	5 (7.6)	2 (2.9)	3 (2.9)	2 (5.4)	12 (4.3)	9 (6.0)	13 (8.3)	25 (16.9)	17 (12.3)	64 (10.8)	0.001
9N	0 (0.0)	1 (1.4)	1 (1.0)	0 (0.0)	2 (0.7)	1 (0.7)	3 (1.9)	1 (0.7)	2 (1.4)	7 (1.2)	0.727
11A	0 (0.0)	0 (0.0)	1 (1.0)	0 (0.0)	1 (0.4)	0 (0.0)	2 (1.3)	3 (2.0)	1 (0.7)	6 (1.0)	0.441
12F	1 (1.5)	1 (1.4)	1 (1.0)	0 (0.0)	4 (1.4)	0 (0.0)	2 (1.3)	2 (1.4)	0 (0)	4 (0.7)	0.686
**CAP due to *S. pneumoniae***	21 (31.8)	15 (21.4)	36 (34.6)	14 (37.8)	86	46 (30.5)	50 (31.8)	57 (38.5)	54 (39.1)	207 (34.9)	
PCV13 serotypes	11 (52.4)	9 (60.0)	20 (55.6)	7 (50.0)	47 (54.6)	19 (41.3)	22 (44.0)	10 (17.5)	15 (27.8)	66 (31.9)	0.004
PCV15 serotypes	11 (52.4)	10 (66.7)	23 (69.9)	7 (50.0)	51 (59.3)	20 (43.5)	22 (44.0)	11 (19.3)	15 (27.8)	68 (32.9)	0.002
PCV20 serotypes	17 (81.0)	13 (86.7)	29 (80.6)	9 (64.3)	68 (79.1)	31 (61.4)	40 (80.0)	44 (77.2)	33 (61.1)	148 (71.5)	0.180
PPSV23 serotypes	17 (81.0)	14 (93.3)	30 (83.3)	10 (71.4)	71 (82.6)	33 (71.7)	44 (88.0)	44 (77.2)	35 (64.8)	156 (75.4)	0.179
**Non-invasive CAP ^1^**	12 (57.1)	11 (73.3)	28 (77.8)	13 (92.9)	64 (74.4)	41 (89.1)	45 (90.0)	46 (80.7)	45 (83.3)	177 (85.5)	
PCV13 serotypes	7 (58.3)	8 (72.7)	15 (53.6)	6 (46.2)	36 (56.3)	18 (43.9)	22 (48.9)	10 (21.7)	13 (28.9)	63 (35.6)	0.004
PCV15 serotypes	7 (58.3)	8 (72.7)	17 (60.7)	6 (46.2)	38 (59.4)	19 (46.3)	22 (48.9)	11 (23.9)	13 (28.9)	65 (36.7)	0.002
PCV20 serotypes	11 (91.7)	10 (90.9)	21 (75.0)	8 (61.5)	50 (78.1)	27 (65.9)	36 (80.0)	34 (73.9)	24 (53.3)	121 (68.4)	0.140
PPSV23 serotypes	11 (91.7)	10 (90.9)	22 (78.6)	9 (69.2)	52 (78.1)	29 (70.7)	39 (86.7)	34 (73.9)	26 (57.8)	128 (72.3)	0.159
**Invasive CAP ^2^**	9 (42.9)	4 (26.7)	8 (22.2)	1 (7.1)	22 (25.6)	5 (10.9)	5 (10.0)	11 (19.3)	9 (16.7)	30 (14.5)	
PCV13 serotypes	4 (44.4)	1 (25.0)	5 (62.5)	1 (100.0)	11 (50.0)	1 (20.0)	0 (0.0)	0 (0.0)	2 (22.2)	3 (10.0)	0.001
PCV15 serotypes	4 (44.4)	2 (50.0)	6 (75.0)	1 (100.0)	13(59.1)	1 (20.0)	0 (0.0)	0 (0.0)	2 (22.2)	3 (10.0)	<0.001
PCV20 serotypes	6 (66.7)	3(75.0)	8 (100.0)	1 (100.0)	18 (81.8)	4 (80.0)	4 (80.0)	10 (90.9)	9 (100.0)	27 (90.0)	0.393
PPSV23 serotypes	6 (66.7)	4 (100.0)	8 (100.0)	1 (100.0)	19 (86.4)	4 (80.0)	5 (100.0)	10 (90.9)	9 (100.0)	27 (90.0)	0.400

^1^ Confirmed pneumococcal CAP (according to UAD1/UAD2 tests or BinaxNow^®^) for which blood and/or pleural fluid culture results were negative; ^2^ *S. pneumoniae* isolated in blood and/or pleural fluid culture. Among 52 cases identified, 1 isolate was not serotyped. * Calculated from the total number of cases by center location. CAP: community-acquired pneumonia; PCV13: 13-valent pneumococcal conjugate vaccine; PCV15: 15-valent pneumococcal conjugate vaccine; PCV20: 20-valent pneumococcal conjugate vaccine; PPSV23: 23-valent pneumococcal polysaccharide vaccine.

## Data Availability

The data presented in this study are available on request from the corresponding author.

## Supplementary Materials

The following supporting information can be downloaded at https://www.mdpi.com/article/10.3390/microorganisms11112781/s1, Supplementary methods: Inclusion criteria, Microbiological studies, Supplementary Figure S1: Flow chart of study patients and sample collection, 2016–2020; Supplementary Figure S2: Microbiological results of CAP (*N* = 1948) and tests used for the diagnosis of pneumococcal pneumonia (*N* = 680), 2016–2020; Supplementary Table S1: Serotype distribution by the presence of complicated pneumonia at hospital admission; Supplementary Table S2: Serotype distribution in all CAP according to number of underlying conditions, 2016–2020; Supplementary Table S3: Serotype distribution in all CAP and pneumococcal CAP according to the presence of underlying conditions by age group, 2016–2020; Supplementary Table S4: Serotype distribution by presence of complications during hospitalization, 2016–2020; Supplementary Table S5: Serotype distribution by ICU admission, 2016–2020. References [10,19,20,21,22,23] are cited in the Supplementary Materials.

## Appendix A

CAPA study group. Hospital Clínic (Barcelona, Spain): A. Torres, C. Cilloniz, A. Ceccato, A. San José, L. Bueno, F. Marco; Hospital Universitario y Politécnico La Fe (Valencia, Spain): R. Menéndez, R. Méndez, I. Amara, A. Gil-Brusola, P. González, A. Latorre; Hospital Galdakao-Usansolo (Galdácano, Spain): P.P. España, M. Egurrola, A. Uranga, A.P. Martínez de la Fuente, A. Artaraz, N. Pérez; Hospital Alvaro Cunqueiro (Vigo, Spain): A. Fernández-Villar, M. Botana-Rial, F. Vasallo, A. Priegue; Biodonostia, Hospital Universitario Donostia (San Sebastián, Spain): J.M. Marimón, E. Pérez-Trallero, M. Ercibengoa; Pfizer S.L.U (Madrid, Spain): C. Méndez, I. Cifuentes, C. Balseiro, J. Sáez, A. Perianes, A. Díaz, E. Garijo, E. Fernández, J. Martínez, R. Pérez, F. Jiménez, M. González, A. Pérez, M. Riera and M.L. Samaniego.
