# Supplementary material for: Pneumococcal Serotypes Associated with Community-Acquired Pneumonia Hospitalizations in Adults in Spain, 2016–2020: The CAPA Study
Source: Microorganisms. 2023 Nov 16;11(11):2781. doi: 10.3390/microorganisms11112781 (PMC10673231; doi:10.3390/microorganisms11112781)
Supplement: Supplementary file 1 [file microorganisms-11-02781-s001.zip › microorganisms-2694719-supplementary.pdf]

Supplementary Material

Methods

Inclusion criteria

As described elsewhere [10], patients were included if they were 18 years old or older, hospitalized in one of the study sites with a diagnosis of confirmed CAP (defined as presence of a new infiltrate on chest radiography accompanied by acute signs and symptoms suggestive of lower respiratory tract infection), immunocompetent and provided written informed consent to participate in the study. Patients were excluded if they had a hospital stay of 48 h or longer in the preceding 15 days, were immunosuppressed (due to AIDS/HIV infection or any other immunosuppressive treatment or condition other than immunosenescence), had sickle cell anaemia or active pulmonary tuberculosis, were receiving current cancer treatment or had received a cancer treatment during the year before the study, or had functional or anatomic asplenia.

Microbiological studies

Pneumococcal isolates were identified by the optochin susceptibility and bile solubility tests. They were stored frozen in skimmed milk at ≤ -40°C until they were delivered to the local central laboratory (Microbiology Department, University Hospital Donostia, Donostia-San Sebastián, Spain). Serotyping was performed by multiplex-PCR [20] and confirmed by the Quellung reaction using polyclonal antisera (Statens Serum Institute, Copenhagen, Denmark). Antibiotic susceptibility testing (broth microdilution, CLSI guidelines) and genotyping (MLST) were performed by the central laboratory on all isolates received.

Urine samples were obtained for all patients (n= 1948) and sent to the sponsor's central laboratory in Pearl River, New York, USA for serotype specific urinary antigen detection (UAD) testing. Unlike other procedures, which were performed per standard of care, the UAD test was a protocol requirement to be performed on the urine samples collected from every

patient included in the study. The assay consists of an immunochromatographic membrane assay using monoclonal antibodies of the serotype-specific capsular polysaccharide. UAD1 detects the thirteen *S. pneumoniae* serotypes included in PCV13 (serotypes 1, 3, 4, 5, 6A, 6B, 7F, 9V, 14, 18C, 19A, 19F and 23F) (UAD1) [22] and the UAD2, introduced in 2016, identifies 11 additional serotypes (2, 8, 9N, 10A, 11A, 12F, 15B, 17F, 20, 22F, 33F) (i.e., all serotypes in 23-valent pneumococcal polysaccharide vaccine [PPSV23] and not included in UAD1) [23].

Figures

Figure S1. Flow chart of study patients and sample collection, 2016-2020

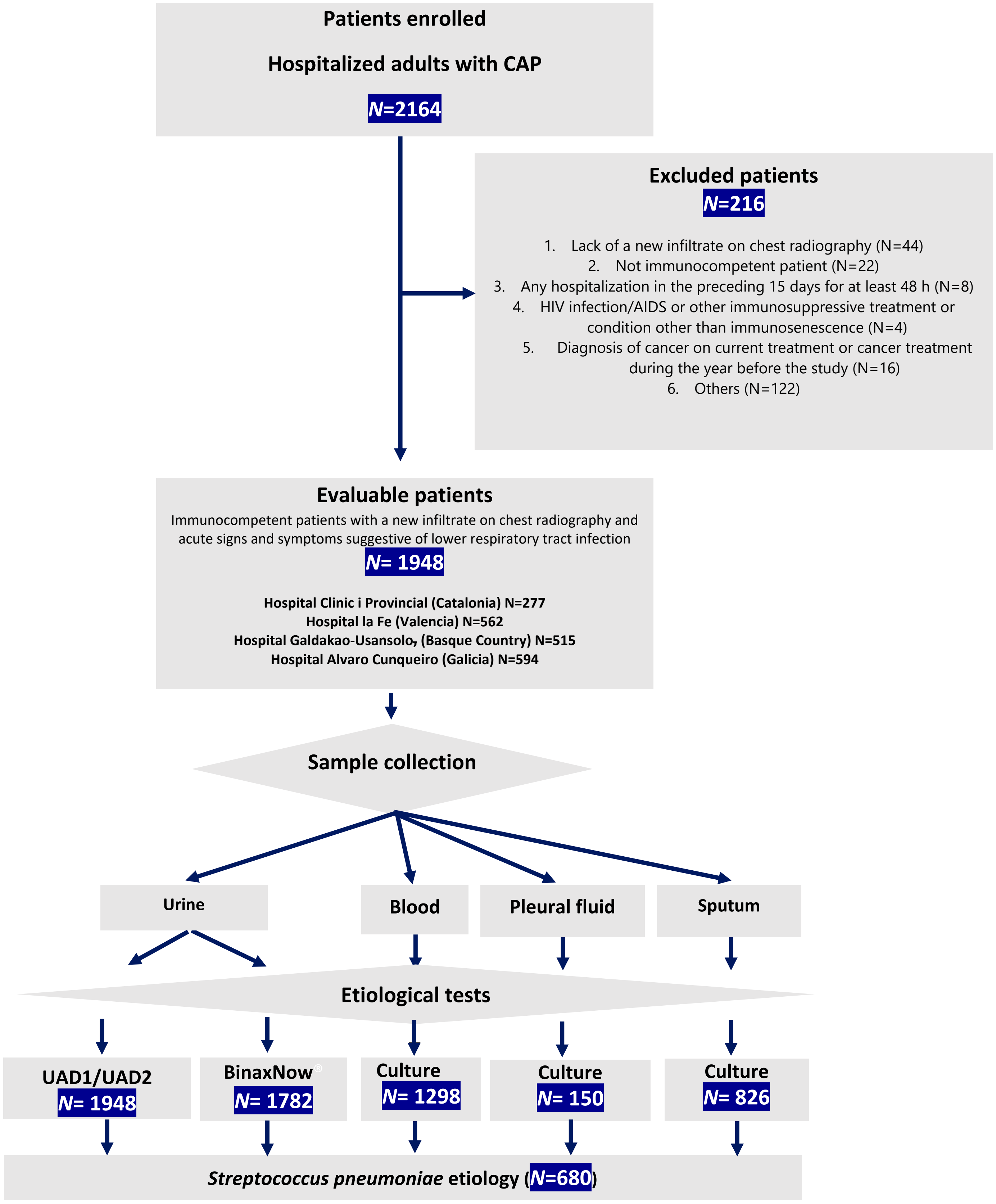

AIDS: acquired immunodeficiency syndrome; HIV: human immunodeficiency virus; UAD1/2: urinary antigen detection test 1 and 2

Figure S2. Microbiological results of CAP (N=1948) and tests used for the diagnosis of pneumococcal pneumonia (N=680), 2016-2020

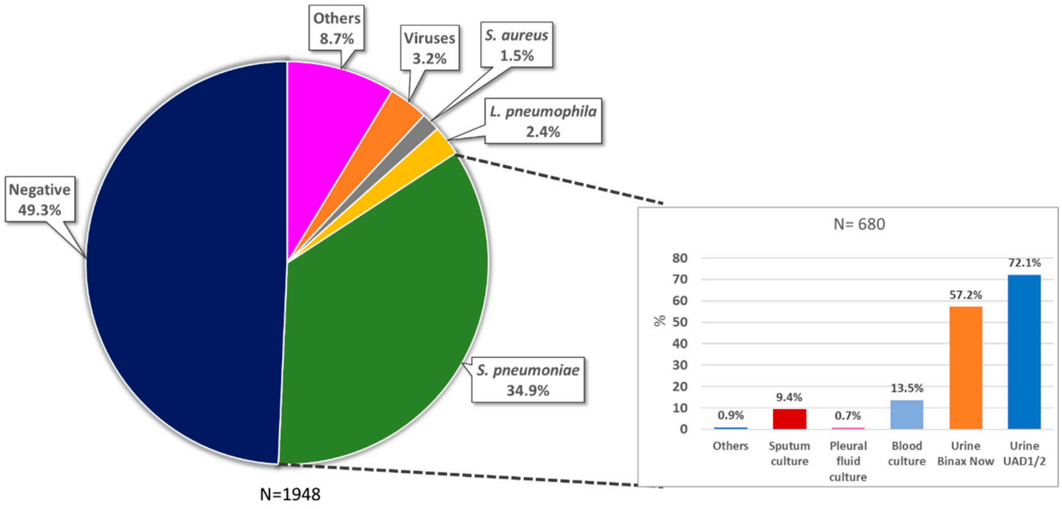

CAP: community-acquired pneumonia; UAD1/2: serotype specific urinary antigen detection test 1 and 2

Supplementary Tables

Table S1. Serotype distribution by presence of complicated pneumonia at hospital admission

	**Non-complicated pneumonia**		**Complicated pneumonia**		***p- value***
	N	**%**	***N***	**%**	
**All CAP**	463		1482		-
PCV13 serotypes	37	8.0	198	13.4	0.002
PCV15 serotypes	47	10.2	220	14.8	0.010
PCV20 serotypes	93	20.1	372	25.1	0.027
PPSV23 serotypes	95	20.5	392	26.5	0.010
**Most prevalent serotypes (≥ 1% in total cases)**					
3	23	5.0	129	8.7	0.009
8	37	8.0	116	7.8	0.909
14	3	0.6	17	1.1	0.439
19A	1	0.2	17	1.1	0.092
9N	5	1.1	16	1.1	
22F	7	1.5	15	1.0	0.448
11A	6	1.3	11	0.7	0.260
**CAP due to *S. pneumoniae***	137		543		
PCV13 serotypes	37	27.0	198	36.5	0.038
PCV15 serotypes	47	34.3	220	40.5	0.184
PCV20 serotypes	93	67.9	372	68.5	0.888
PPSV23 serotypes	95	69.3	392	72.2	0.509
**Non-invasive CAP[1]**	129		450		
PCV13 serotypes	37	28.7	173	38.4	0.042
PCV15 serotypes	47	36.4	190	42.2	0.239
PCV20 serotypes	85	65.9	297	66.0	0.982
PPSV23 serotypes	87	67.4	310	68.9	0.755
**Invasive CAP[2]**	**8**		**93**		
PCV13 serotypes	0	0.0	25	26.9	0.091
PCV15 serotypes	0	0.0	30	32.3	0.055
PCV20 serotypes	8	100.0	75	80.6	0.170
PPSV23 serotypes	8	100.0	82	88.2	0.303

1. Confirmed pneumococcal CAP (by UAD1/UAD2 tests or BinaxNow®) for which blood and/or pleural fluid culture result were negative; 2. Isolate of *S. pneumoniae* in blood and/or pleural fluid. Among 101 cases identified, 4 isolates were not serotyped.

CAP: community-acquired pneumonia; PCV13: 13-valent pneumococcal conjugate vaccine; PCV15: 15-valent pneumococcal conjugate vaccine; PCV20: 20-valent pneumococcal conjugate vaccine; PPSV23: 23-valent pneumococcal polysaccharide vaccine.

Table S2. Serotype distribution in all CAP according to number of underlying conditions, 2016-2020.

	**No comorbidities**		**≥1 comorbidity**		***p-value***
	N	**%**	***N***	**%**	
All CAP	295		1652		
PCV13 serotypes	29	9.8	206	12.5	0.544
PCV15 serotypes	33	11.2	234	14.2	0.296
PCV20 serotypes	71	24.1	394	23.8	0.544
PPSV23 serotypes	74	25.1	413	25.0	0.127

CAP: community-acquired pneumonia; PCV13: 13-valent pneumococcal conjugate vaccine; PCV15: 15-valent pneumococcal conjugate vaccine; PCV20: 20-valent pneumococcal conjugate vaccine; PPSV23: 23-valent pneumococcal polysaccharide vaccine

Table S3. Serotype distribution in all CAP and in pneumococcal CAP according to presence of underlying conditions by age group, 2016-2020[a]

		Diabetes mellitus		COPD		Previous pneumonia		Tobacco cigarette smoking		Chronic heart failure		Chronic renal failure		Asthma		No underlying diseases	
		N	%	*N*	%	*N*	%	*N*	%	*N*	%	*N*	%	*N*	%	N	%
		**18-64 years**															
**All CAP (n = 801)**		83		66		117		283		23		10		106		193	
	**PCV13 serotypes**	11	13.3	10	15.2	9	7.7	32	11.3	2	8.7	0	0.0	10	9.4	17	8.8
	**PCV15 serotypes**	13	15.7	12	18.2	9	7.7	34	10.0	2	8.7	0	0.0	11	10.4	21	10.9
	**PCV20 serotypes**	21	25.3	24	36.4	21	17.9	84	29.7	3	13.0	2	20.0	30	28.3	50	25.9
	**PPSV3 serotypes**	24	28.9	24	36.4	22	18.8	87	30.7	4	17.4	2	20.0	31	29.2	51	26.4
**Most prevalent serotypes (≥1% in total cases)**	**23F**	1	1.2	0	0.0	0	0.0	1	.4	0	0.0	0	0.0	0	0.0	0	0.0
	**14**	0	0.0	1	2.6	0	0.0	3	2.1	0	0.0	0	0.0	0	0.0	0	0.0
	**19A**	1	2.2	1	2.6	1	1.5	1	0.7	1	7.7	0	0.0	2	4.2	0	0.0
	**3**	7	8.4	8	12.1	7	6.0	18	6.4	1	4.3	0	0.0	6	5.7	9	4.7
	**7F**	0	0.0	0	0.0	0	0.0	1	.4	0	0.0	0	0.0	0	0.0	3	1.6
	**8**	7	8.4	8	12.1	11	9.4	41	14.5	1	4.3	2	20.0	17	16.0	24	12.4
	**12F**	1	1.2	2	30.0	0	0.0	5	1.8	0	0.0	0	0.0	1	0.9	4	2.1
	**9N**	3	3.6	0	0.0	2	1.7	3	1.1	0	0.0	0	0.0	2	1.9	1	0.5
	**20**	0	0.0	0	0.0	0	0.0	0	0.0	1	4.3	0	0.0	0	0.0	0	0.0
	**22F**	2	2.4	2	30.0	0	0.0	2	0.7	0	0.0	0	0.0	1	0.9	3	1.6
	**11A**	0	0.0	1	1.5	1	0.9	3	1.1	0	0.0	0	0.0	0	0.0	1	0.5
	**10A**	0	0.0	1	1.5	0	0.0	1	0.4	0	0.0	0	0.0	0	0.0	0	0.0
	**7F**	0	0.0	0	0.0	0	0.0	1	0.4	0	0.0	0	0.0	0	0.0	3	1.6
	**4**	1	1.2	0	0.0	0	0.0	4	1.4	0	0.0	0	0.0	1	.9	0	0.0
	**5**	1	1.2	0	0.0	0	0.0	1	0.4	0	0.0	0	0.0	0	0.0	0	0.0
		**≥ 65 years**															
**All CAP (N = 1147)**		340		285		243		122		160		158		98		102	
	**PCV13 serotypes**	33	9.7	33	11.6	24	9.9	20	16.4	15	9.4	10	6.3	17	17.3	12	11.8
	**PCV15 serotypes**	36	10.6	38	13.3	30	12.3	26	21.3	17	10.6	10	6.3	19	19.4	12	11.8
	**PCV20 serotypes**	61	17.9	58	20.4	43	17.7	40	32.8	21	13.1	19	12.0	25	25.5	21	20.6
	**PPSV3 serotypes**	71	20.9	66	23.2	47	19.3	43	35.2	22	13.8	18	11.4	23	23.5	23	22.5

**Most prevalent serotypes (≥ 1% in total cases)**	**23F**	0	0.0	1	0.4	1	0.4	0	0.0	0	0.0	0	0.0	0	0.0	1	1.0
	**14**	5	1.5	1	0.4	0	0.0	0	0.0	0	0.0	2	1.3	3	3.1	0	0.0
	**18C**	0	0.0	0	0.0	1	0.4	1	0.8	0	0.0	0	0.0	1	1.0	0	0.0
	**19A**	1	0.3	1	0.4	0	0.0	3	2.5	0	0.0	0	0.0	0	0.0	1	1.0
	**19F**	0	.0	1	0.4	0	0.0	0	0.0	2	1.3	0	0.0	0	0.0	0	0.0
	**3**	25	7.4	23	8.1	21	8.6	13	10.7	9	5.6	5	3.2	11	11.2	9	8.8
	**7F**	1	0.6	1	0.6	0	0.0	0	0.0	0	0.0	0	0.0	0	0.0	0	0.0
	**8**	25	7.4	23	8.1	21	8.6	13	10.7	9	5.6	5	3.2	11	11.2	9	8.8
	**12F**	0	0.0	1	0.4	0	0.0	0	0.0	0	0.0	2	1.3	0	0.0	0	0.0
	**9N**	6	1.8	6	2.1	2	0.8	4	3.3	1	0.6	1	0.6	0	0.0	1	1.0
	**22F**	2	0.6	3	1.1	4	1.6	4	3.3	1	0.6	0	0.0	0	0.0	0	0.0
	**11A**	3	0.9	5	1.8	2	0.8	3	2.5	0	.0	1	.6	0	0.0	1	1.0
	**10A**	2	0.6	2	0.7	1	0.4	1	0.8	1	0.6	0	0.0	0	0.0	1	1.0
	**4**	0	0.0	3	1.1	0	0.0	1	0.8	2	1.3	1	0.6	0	0.0	0	0.0

a. Patients might have more than one underlying condition. CAP: community-acquired pneumonia; COPD: chronic obstructive pulmonary disease; PCV13: 13-valent pneumococcal conjugate vaccine; PCV15: 15-valent pneumococcal conjugate vaccine; PCV20: 20-valent pneumococcal conjugate vaccine; PPSV23: 23-valent pneumococcal polysaccharide vaccine.

Table S4. Serotype distribution by presence of complications during hospitalization, 2016-2020.

	**No complications**		**Complications**		***p- value***
	N	**%**	***N***	**%**	
**All CAP**	**1248**		**700**		
PCV13 serotypes	130	10.4	105	15.0	0.003
PCV15 serotypes	155	12.4	112	16.0	0.027
PCV20 serotypes	271	21.7	194	27.7	0.003
PPSV23 serotypes	285	22.8	202	28.9	0.003
**Most prevalent serotypes (≥ 1% in total cases)**					
3	82	6.6	70	10.0	0.007
8	90	7.2	63	9.0	0.159
19A	9	0.7	9	1.3	0.224
14	12	1.0	8	1.1	0.815
12F	10	0.8	7	1.0	0.623
9N	15	1.2	6	0.9	0.648
11A	12	1.0	5	0.7	0.800
22F	17	1.4	5	0.7	0.264
**CAP due to *S. pneumoniae***	424		256		
PCV13 serotypes	130	30.7	105	41.0	0.006
PCV15 serotypes	155	36.6	112	43.8	0.063
PCV20 serotypes	271	63.9	194	75.8	0.001
PPSV23 serotypes	285	67.2	202	78.9	0.001
**Non-invasive CAP[1]**	**369**		**210**		
PCV13 serotypes	116	31.4	94	44.8	0.001
PCV15 serotypes	138	37.4	99	47.1	0.022
PCV20 serotypes	227	61.5	155	73.8	0.003
PPSV23 serotypes	238	64.5	159	75.7	0.005
**Invasive CAP[2]**	**55**		**46**		
PCV13 serotypes	14	25.5	11	23.9	0.858
PCV15 serotypes	17	30.9	13	28.3	0.772
PCV20 serotypes	44	80.0	39	84.8	0.532
PPSV23 serotypes	47	85.5	43	93.5	0.197

1. Confirmed pneumococcal CAP (by UAD1/UAD2 or BinaxNow® tests) for which blood and/or pleural fluid culture result were negative; 2. Isolate of *S. pneumoniae* in blood and/or pleural fluid. Four isolates of invasive pneumococcal pneumonias were not serotyped due to lack of sample or lysis. CAP: community-acquired pneumonia; PCV13: 13-valent pneumococcal conjugate vaccine; PCV15: 15-valent pneumococcal conjugate vaccine; PCV20: 20-valent pneumococcal conjugate vaccine; PPSV23: 23-valent pneumococcal polysaccharide vaccine.

Table S5. Serotype distribution by ICU admission, 2016-2020.

	No admission to ICU		ICU admission		*p- value*
	N	**%**	***N***	**%**	
**All CAP**	1729		218		
PCV13 serotypes	185	10.7	50	22.9	<0.001
PCV15 serotypes	214	12.4	53	24.3	<0.001
PCV20 serotypes	390	22.6	75	34.4	<0.001
PPSV23 serotypes	411	23.8	76	34.9	<0.001
**Most prevalent serotypes (≥ 1% in total cases)**					
3	116	6.7	36	16.5	<0.001
8	138	8.0	15	6.9	0.569
14	15	0.9	5	2.3	0.064
12F	13	0.8	4	1.8	0.113
19A	15	0.9	3	1.4	0.444
22F	20	1.2	2	0.9	1.000
9N	20	1.2	1	0.5	0.502
**CAP due to *S. pneumoniae***	588		92		
PCV13 serotypes	185	31.5	50	54.3	<0.001
PCV15 serotypes	214	36.4	53	57.6	<0.001
PCV20 serotypes	390	66.3	75	81.5	0.004
PPSV23 serotypes	411	69.9	76	82.6	0.012
**Non-invasive CAP[1]**	515		64		
PCV13 serotypes	174	33.8	36	56.3	<0.001
PCV15 serotypes	199	38.6	38	59.4	0.001
PCV20 serotypes	330	64.1	52	81.3	0.006
PPSV23 serotypes	345	67.0	52	81.3	0.020
**Invasive CAP[2]**	73		28		
PCV13 serotypes	11	15.1	14	50.0	<0.001
PCV15 serotypes	15	20.5	15	53.6	0.001
PCV20 serotypes	60	82.2	23	82.1	0.995
PPSV23 serotypes	66	90.4	24	85.7	0.495

1. Confirmed pneumococcal CAP (by UAD1/UAD2 or BinaxNow® tests) for which blood and/or pleural fluid culture result were negative; 2. Isolate of S. pneumoniae in blood and/or pleural fluid. Four isolates of invasive pneumococcal pneumonias were not serotyped due to lack of sample or lysis. CAP: community-acquired pneumonia; ICU: intensive care unit; PCV13: 13-valent pneumococcal conjugate vaccine; PCV15: 15-valent pneumococcal conjugate vaccine; PCV20: 20-valent pneumococcal conjugate vaccine; PPSV23: 23-valent pneumococcal polysaccharide vaccine.
